# Unexpected haemorrhage from lateral thoracic artery following the removal of a pleural drainage tube

**DOI:** 10.1002/rcr2.882

**Published:** 2021-11-23

**Authors:** Keigo Matsuura, Makoto Tada, Toshiyuki Sumi, Koichi Osuda, Hisashi Nakata, Hirofumi Chiba

**Affiliations:** ^1^ Department of Pulmonary Medicine Hakodate Goryoukaku Hospital Hakodate Japan; ^2^ Department of Respiratory Medicine and Allergology Sapporo Medical University School of Medicine Sapporo Japan; ^3^ Department of Thoracic Surgery Hakodate Goryoukaku Hospital Hakodate Japan; ^4^ Division of Radiology Hakodate Goryoukaku Hospital Hakodate Japan

**Keywords:** haemorrhage, lateral thoracic artery, pneumothorax

## Abstract

Pulmonologists must be aware of the possible arterial bleeding even during the removal of a safely inserted drain.

## Graphical abstract

We report an unexpected haemorrhage from lateral thoracic artery following the removal of a pleural drainage tube which had been safely inserted. Pulmonologists must be aware of the possible arterial bleeding even during the removal of a safely inserted drain.

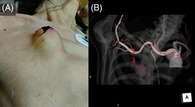

## CLINICAL IMAGE

**FIGURE 1:**
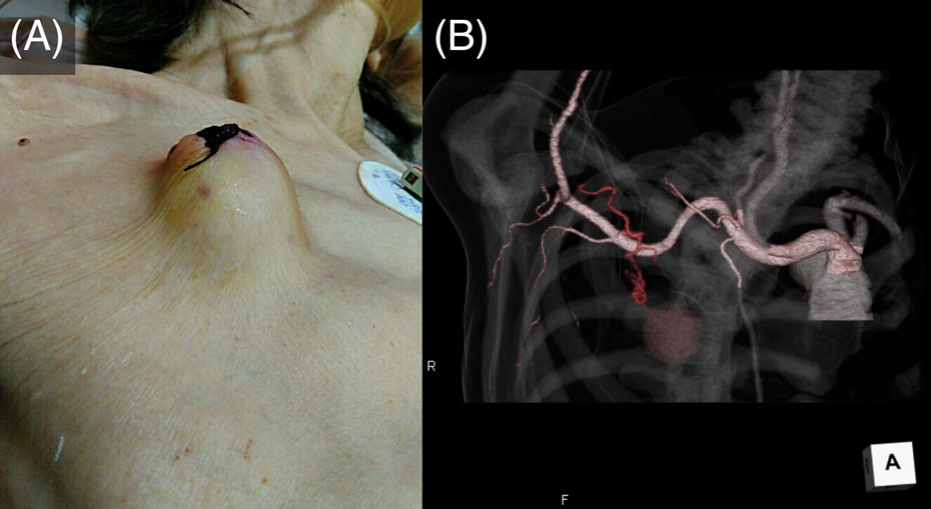
(A) After removal of the thoracic drainage tube, a subcutaneous haematoma developed at the removal site. (B) Volume rendering reconstruction shows the inflow of the lateral thoracic artery into the subcutaneous haematoma

A 65‐year‐old woman developed secondary pneumothorax following interstitial pneumonia. As computed tomography showed pleural adhesions, a drainage tube was inserted, without bleeding complications, under ultrasound guidance via the right second intercostal space of the anterior chest within a safe space. The patient had refractory pneumothorax with air leaks for 2 months. The air leaks resolved after multiple autologous blood patch procedures. Pulsatile bleeding was observed from the drainage removal site after the drainage tube was removed. Although pressure haemostasis was achieved by suturing, a mass was formed at the site (Figure 1A). Contrast‐enhanced computed tomography showed inflow from the lateral thoracic artery (Figure 1B); the vessel was therefore surgically ligated under local anaesthesia. Among the complications associated with thoracic drainage, bleeding has an incidence of 4.9%^1^; however, serious bleeding is rare. Only one report has described bleeding after drainage tube removal, which was caused by intercostal artery injury.^2^ Extensive pleural adhesions led physicians to insert drainage tube through an anterior intercostal space, thus increasing the risk of vascular damage. In this case, bleeding may have been caused by angiogenesis at the insertion site during wound healing or vessel injury during drain removal. Pulmonologists must be aware of the possible arterial bleeding even during the removal of a safely inserted drain.

## CONFLICT OF INTEREST

None declared.

## AUTHOR CONTRIBUTION

Toshiyuki Sumi conceived the idea for the manuscript and drafted it. Keigo Matsuura and Makoto Tada contributed to the clinical management of the patient. Koichi Osuda and Hisashi Nakata were responsible for radiographic diagnosis. Hirofumi Chiba revised the manuscript for intellectual content. All authors contributed to and approved the final version of the manuscript.

## ETHICS STATEMENT

The authors declare that appropriate written informed consent was obtained for the publication of this manuscript and accompanying images.
